# High-resolution genetic maps and QTL mapping applications reveal *polycystin* gene involvement in oyster shell formation

**DOI:** 10.1016/j.isci.2025.113986

**Published:** 2025-11-10

**Authors:** Haitao Ma, Yanping Qin, Dongmei Yu, Bingke Jiao, Qingliang Liao, Yang Zhang, Yinjie Zhang, Jingyue Huang, Jun Li, Ziniu Yu, Yuehuan Zhang

**Affiliations:** 1 Key Laboratory of Breeding Biotechnology and Sustainable Aquaculture (CAS), Key Laboratory of Tropical Marine Bio-resources and Ecology, Guangdong Provincial Key Laboratory of Applied Marine Biology, South China Sea Institute of Oceanology, Chinese Academy of Sciences, Guangzhou 510301, China; 2 Sanya Marine Eco-environment Engineering Research Institute, Tropical Marine Biological Research Station in Hainan, Chinese Academy of Sciences, Sanya 572024, China; 3 Southern Marine Science and Engineering Guangdong Laboratory (Zhuhai), Zhuhai 519015, China; 4 Daya Bay Marine Biology Research Station, Chinese Academy of Sciences, Shenzhen 518124, China; 5 Agro-Tech Extension Center of Guangdong Province, Guangzhou, China; 6 Shenzhen Institute of Guangdong Ocean University, Shenzhen, China; 7 Guangdong Key Laboratory of Animal Conservation and Resource Utilization, Guangdong Public Laboratory of Wild Animal Conservation and Utilization, Institute of Zoology, Guangdong Academy of Sciences, Guangzhou, China; 8 State Key Laboratory of Plant Genomics, Institute of Genetics and Developmental Biology, Innovation Academy for Seed Design, Chinese Academy of Sciences, Beijing 100101, China; 9 University of Chinese Academy of Sciences, Beijing 100049, China

**Keywords:** Marine organism, Developmental biology, Genomic analysis, Sequence analysis

## Abstract

Genetic maps are efficient methods to study the molecular mechanisms of introgression and hybridization. The high-resolution genetic maps were constructed for *Crassostrea hongkongensis* and *C. sikamea* with reciprocal hybrid families. Thirty-five QTLs and twenty genes for shell height and shell length were identified, in which the *polycystin* gene was chosen for systematic study due to the highest LOD and phenotypic variance contribution. The *polycystin* expressions in two oyster species were highest at the blastula stage and upper mantle. *In situ* hybridization showed that *polycystins* were primarily found in the outer and middle folds of the mantle. Furthermore, the *polycystin* expression reached its highest peak at 24 h (*C. hongkongensis*) or 48 h (*C. sikamea*) after shell notch. The RNAi of *polycystin* resulted in sparse and disordered mineral layers and downregulation of other calcium-regulated genes. Conclusively, these results provided experimental support for the *polycystin* participating in shell formation and offered valuable molecular targets for further genetic analysis.

## Highlights

• The high-resolution genetic maps were constructed for the two brackish oysters
• Thirty-five QTLs and twenty genes for shell height and shell length were identified
• *Polycystin* chosen for this study due to highest LOD and phenotypic variance contribution
• Functional experiments confirmed *Polycystin* involvement in oyster shell formation

## Graphical abstract

**Figure:**
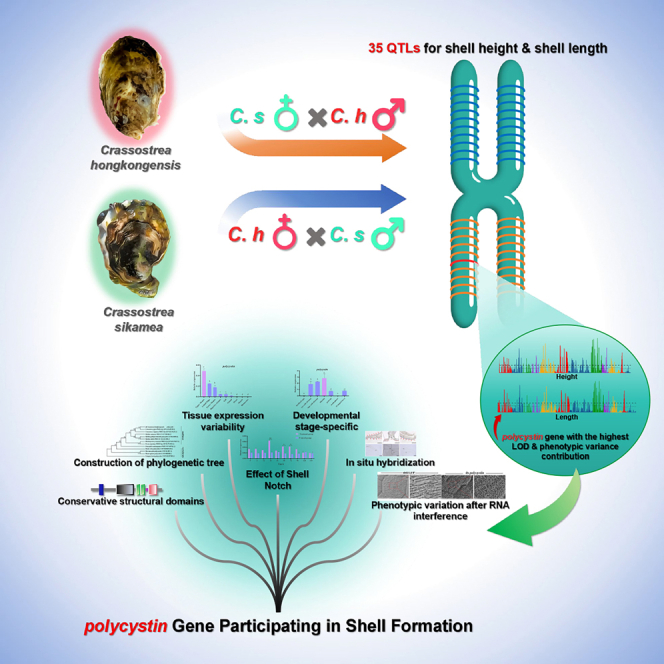

## Introduction

Oysters are an important aquaculture species in China, accounting for about 35% of the total marine mollusk production (5.4 million tons in 2021).^1^ At least five *Crassostrea* oyster species are naturally distributed along the coastal areas of China: *Crassostrea hongkongensis* (Hong Kong oyster), *C*. *ariakensis* (Jinjiang oyster), *C*. *sikamea* (Kumamoto oyster), *C*. *angulata* (Portuguese oyster), and *C*. *gigas* (Pacific oyster).^2^ Oysters can be divided into two major groups based on their natural habitats: seawater oysters (*C. angulata* and *C. gigas*) and brackish oysters (*C. sikamea*, *C. arikensis*, *C. hongkongensis*).^3^

*C. hongkongensis* and *C. sikamea* share the same habitats and geographical distribution.^4^ The aquaculture history of *C. hongkongensis* dates back nearly 1000 years, and its annual production now exceeds 1.6 million metric tons.^5^ *C. sikamea* is also an important wild fishery resource, distributed on stones and reefs in the inter-tidal zone of the South China Sea, ranging from Jiangsu to Hainan provinces.^5^^,^^6^ To advance oyster genetic improvement, reciprocal hybrids were bred through two-way hybridization, resulting in oysters fully adapted to brackish environments with higher growth rate (compared to *C. sikamea*) and improved flesh quality.^5^

High-density genetic maps are powerful tools for studying the molecular mechanisms of introgression and hybridization. In genetic maps, reduced gene flow can be identified through the limited recombination of rearranged chromosomes.^7^ Moreover, genetic maps are useful for chromosome-scale scaffolding, comparative genomic analysis, and the detection of recombination hotspots. To date, genetic linkage maps have been developed for a few oyster species, including *C*. *virginica*,^8^^,^^9^ *C*. *gigas*,^10^ and *Ostrea edulis*.^11^^,^^12^ Besides, QTL analysis and a high-density SNP genetic linkage map for shell growth-related traits were studied for the first time in a hybrid oyster family (*C. gigas* × *C. angulata*).^13^ However, genetic linkage maps for *C. hongkongensis* and *C. sikamea* remain unavailable.

Genetic mapping can be used to identify quantitative trait loci (QTLs), especially for heterosis, which has been studied in several aquaculture species.^14^ In oysters, genetic studies are often limited by the low number of developed markers and immature high-throughput genotyping platforms. Genotyping by sequencing (GBS) technology is a high-throughput method for fast discovery and genotyping thousands of SNP markers.^15^ This method has been applied in many species for high density linkage map construction and QTL mapping.^13^^,^^16^

In shellfish, shell formation and growth were important aspects of growth traits. In this study, we constructed high-resolution linkage maps using reciprocal F_1_ hybrid families of *C. hongkongensis* and *C. sikamea* at four months of age and conducted QTL analyses for shell growth-related traits. The objectives of this study include: (1) constructing high-resolution genetic maps for *C. hongkongensis* and *C. sikamea*, (2) identifying segregation distortion loci and revealing the genomic incompatibilities between *C. hongkongensis* and *C. sikamea*, (3) constructing QTL fine mapping for shell height and shell length growth and identifying candidate SNP markers and genes related to shell growth; and (4) functionally verifying the key gene through the genetic linkage map, revealing the involvement of the *polycystin* gene in oyster shell formation.

## Results

### Genetic confirmation and characteristics of phenotypic traits

Gel electrophoresis was used to identify hybrid progeny from HS-hybrid family 1 and SH-hybrid family 2. The results indicated a clear band of approximately 328 bp for *C. hongkongensis* parents and 220 bp for *C. sikamea* parents. All progeny from HS-hybrid family 1 and SH-hybrid family 2 indicated two distinct bands at 328 bp and 220 bp, confirming that the hybrid progeny inherited nuclear DNA from both parents and that they are true hybrids.

**Figure 1:**
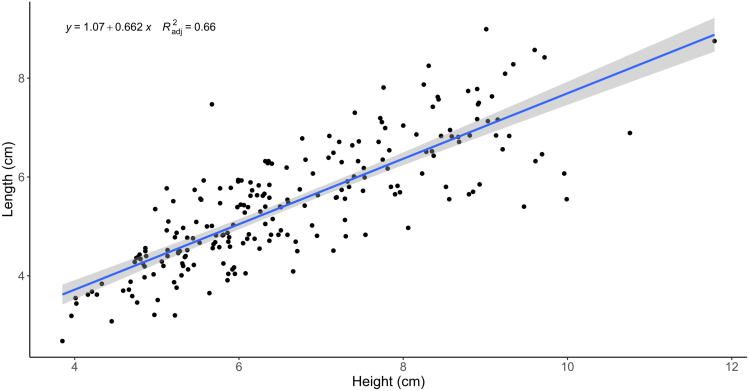
Correlation analysis of two shell growth traits in the hybrid oysters

**Figure 2:**
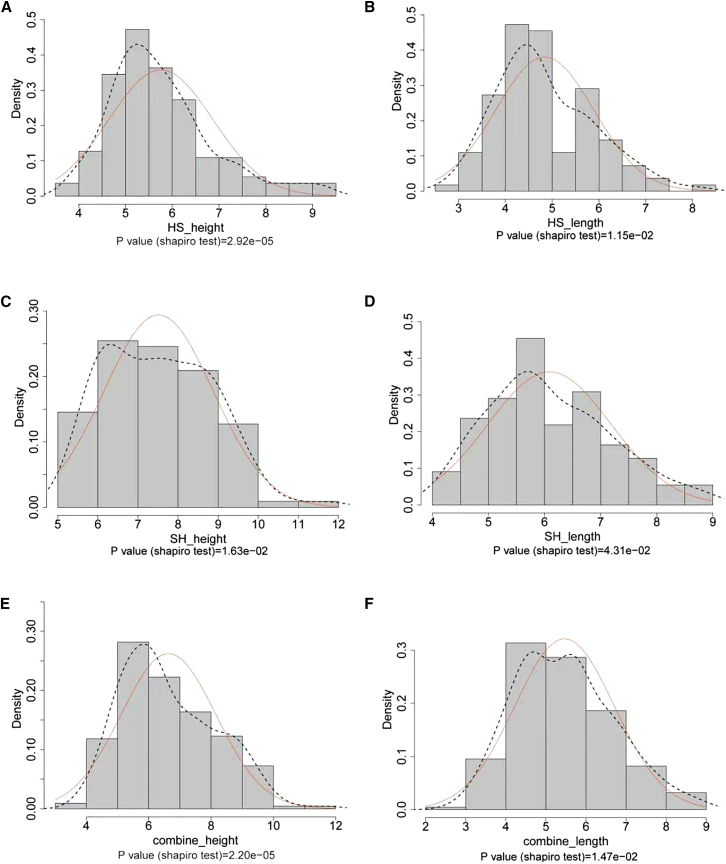
Normality test of measured traits using Shapiro-Wilk test (A) Shell height for HS-hybrid family. (B) Shell length for HS-hybrid family. (C) Shell height for SH-hybrid family. (D) Shell length for SH-hybrid family. (E) Shell height for combined data of HS- and SH-hybrid family. (F) Shell length for combined data of HS- and SH-hybrid family.

The results of the phenotypic analysis of two shell growth traits (shell height and shell length) are shown in Table S2. The average shell height and shell length were 7.52 ± 1.36 cm, 6.09 ± 1.10 cm in HS-hybrid family 1, and 5.75 ± 1.12 cm, 4.84 ± 1.05 cm in SH-hybrid family 2, respectively. These two shell growth traits showed a strong correlation (r = 0.81, *p* < 0.001) (Figure 1). All the measured phenotypic parameters showed a normal distribution, suggesting quantitative inheritance and suitability for QTL mapping (Figure 2). Therefore, all measured phenotype data were used as mapping panels for QTL analysis in *C. hongkongensis* and *C. sikamea*.

### Genotyping by sequencing sequencing and genotyping of the mapping families

Sequencing data were obtained for four parents and 220 progeny from the mapping families. A total of 1571 million reads were generated for the two mapping families. According to the base quality barplot produced by FastQC, bases after position 135 in every R1 read and after position 145 in every R2 reads, were trimmed due to low quality. Sufficient sequencing depth was achieved for the parents' oysters. For the HS-hybrid family 1, a total of 7.0 and 6.7 million high-quality reads were obtained for the *C. hongkongenis* (female) and *C. sikamea* (male) parents, with sequencing depths of 30.4× and 30.0×, respectively. For SH-hybrid family 2, a total of 6.9 and 8.1 million high-quality reads were obtained for the *C. sikamea* (female) and *C. hongkongenis* (male) parents, with sequencing depths of 39.8× and 48.4×, respectively. For the hybrid progeny, about 7.5 million high-quality reads were obtained for each progeny, with a mean sequencing depth of 33.0×, meeting the requirements for accurate genotyping.

The quality of the genome assembly is important for the mapping accuracy of GBS reads. The genome of *C. hongkongenis,* with a genome size of 730 Mb and a contig N50 of 1096 kb, was used for read mapping.^17^ After filtering, 173,330 SNPs remained. The results of the χ^2^ test on the segregation distortion of codominant markers are summarized in Tables 1, S3, and S4. In HS-hybrid family 1, a total of 1607 markers significantly deviated (*p* < 0.05) from the expected Mendelian ratio. In SH-hybrid family 2, a total of 2762 markers showed a significantly different (*p* < 0.05) from the expected Mendelian ratio. Notably, 96.9% and 85.3% of the shared markers were identified. Considering that distorted markers increase the coverage of the genetic map and improve the detection of linked QTL, 847 lm × ll markers and 1370 nn × np markers with segregation ratios between 95:15 and 15:95 (with *p*-value of χ^2^ > 9.49e-15) were retained for HS-hybrid family 1 and 715 lm × ll markers and 3236 nn × np markers with segregation ratios between 90:20 and 20:90 (with *p*-value of χ^2^ > 3.86e-11) were retained for SH-hybrid family 2.

**Table 1:** Genotyping results and marker segregation analysis of the hybrid family

Family	Marker type	Segregation type^a^	Marker number	Distorted marker number^b^	Distored ratio
HS	Codominant	lmxll	947	501	52.9%
		nnxnp	1770	1043	58.9%
		hkxhk	65	63	96.9%
	**Sum**		**2782**	**1607**	**57.8%**
SH	Codominant	lmxll	858	503	58.6%
		nnxnp	3613	2195	60.8%
		hkxhk	75	64	85.3%
	**Sum**		**4546**	**2762**	**60.8%**
**SH+HS**			**7328**	**4369**	**59.6%**

a The segregation type is defined by the parental genotypes. For instance, if the parents have nucleotide genotypes AC and AA, these are recoded as lm and ll, respectively.

b Distorted marker refers to markers exhibiting significant segregation distortion. For this study, markers were classified as distorted if their segregation ratio deviated from the expected Mendelian ratio, specifically outside the ranges of 95:15 to 15:95 in the HS-hybrid family 1 and 90:20 to 20:90 in the SH-hybrid family 2.

### High-resolution linkage mapping for *C. hongkongensis* and *C. sikamea*

**Figure 3:**
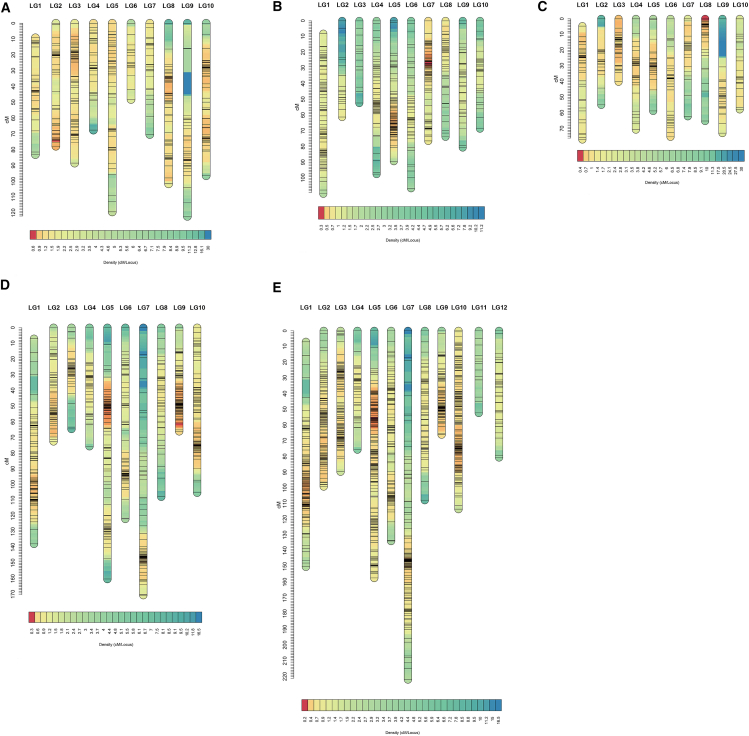
The high-resolution genetic linkage map (A) *C. hongkongensis* containing 583 markers in 10 linkage groups for HS-hybrid family. (B) *C. sikamea* containing 670 markers in 10 linkage groups for HS-hybrid family. (C) *C. hongkongensis* containing 514 markers in 10 linkage groups for SH-hybrid family. (D) *C. sikamea* containing 1149 markers in 10 linkage groups for SH-hybrid family. (E) *C. sikamea* containing 1795 markers in 12 linkage groups for combined of HS- and SH-hybrid family.

In HS-hybrid family 1, 1253 markers in accordance with the segregation ratio were used, grouped into *C. hongkongensis* heterozygous markers (Aa × aa type, 583 markers) and *C. sikamea* heterozygous markers (aa × Aa type, 670 markers). Based on the LOD threshold of 2–15, markers were grouped into 10 linkage groups (LGs), in agreement with the haploid chromosome number of haploid *Crassostrea*.^18^ The linkage map of *C. hongkongensis* consists of 583 markers spanning 875.12 cM (Table 2; Figure 3A), with a mean marker interval of 1.5 cM (range from 1.12 cM in LG3 to 2.4 cM in LG9). According to two different methods (Fishman et al., 2001), the expected map length of *C. hongkongensis* was 890.12 cM (G_e1_) and 908.61 cM (G_e2_). Based on the average value of the two expected map lengths (899.37 cM), the genome coverage was 97.30%. For the *C. sikamea* map, 670 markers were mapped spanning over 815.19 cM (Table 3; Figure 3B). The marker interval ranged from 0.63 cM in LG7 to 3.26 cM in LG3, with a mean marker interval of 1.22 cM. The constructed genome was estimated to be 836.94 cM in length by averaging G_e1_ (827.40 cM) and G_e2_ (846.48 cM) and covering about 97.40% of the whole genome. Due to significant differences between *C. sikamea* and *C. hongkongensis*, insufficient shared markers prevent the integration of the two maps.

**Table 2:** Summary of the linkage map for *C. hongkongensis* of HS-hybrid family

Linkage group	Mapped markers	Distinct positions	Genetic length(cM)	Marker interval(cM)
group_1	50	36	83.065	1.66
group_2	67	43	77.753	1.16
group_3	79	63	88.514	1.12
group_4	39	35	67.567	1.73
group_5	79	49	119.367	1.51
group_6	26	20	48.198	1.85
group_7	28	26	70.37	2.51
group_8	80	65	101.633	1.27
group_9	51	39	122.183	2.4
group_10	84	57	96.473	1.15
Total	583	433	875.123	1.5
Ge1	890.12	–	–	–
Ge2	908.61	–	–	–
Ge	899.37	–	–	–
coverage	97.30%	–	–	–

**Table 3:** Summary of the linkage map for *C. sikamea* of HS-hybrid family

Linkage group	Mapped markers	Distinct positions	Genetic length(cM)	Marker interval(cM)
group_1	105	85	109.914	1.05
group_2	32	18	61.139	1.91
group_3	16	13	52.215	3.26
group_4	73	58	97.444	1.33
group_5	105	84	89.153	0.85
group_6	62	52	106.37	1.72
group_7	121	84	76.323	0.63
group_8	64	49	73.66	1.15
group_9	54	38	80.526	1.49
group_10	38	30	68.453	1.8
Total	670	511	815.197	1.22
Ge1	827.4	–	–	–
Ge2	846.48	–	–	–
Ge	836.94	–	–	–
coverage	97.40%	–	–	–

In SH-hybrid family 2, 1663 markers in accordance with the segregation ratio were used, grouped into *C. sikamea* heterozygous markers (Aa × aa type, 1149) and *C. hongkongensis* heterozygous markers (aa × Aa type, 514 markers). Based on the LOD threshold of 2–15, markers were grouped into 10 linkage groups (LGs). The linkage map of *C. sikamea* included 1149 markers spanning 1081.13 cM (Table 4; Figure 3C), with marker intervals ranging from 0.54 cM in LG9 to 1.64 cM in LG7 and a mean marker interval of 0.94 cM. The expected map length was 1090.53 cM (G_e1_) and 1101.65 cM (G_e2_), with an average of 1096.09 cM, resulting in 98.63% genome coverage. The *C. hongkongensis* map included 514 markers spanning 634.04 cM (Table 5; Figure 3D), with marker intervals ranging from 0.63 cM in LG3 to 1.75 cM in LG10, and a mean marker interval of 1.23 cM. The genome length was estimated at 653.36 cM (average of Ge1: 646.34 cM and Ge2: 660.37 cM), covering 97.04% of the genome. Insufficient shared markers also prevented the construction of consensus maps for the two species.

**Table 4:** Summary of the linkage map for *C. sikamea* of SH-hybrid family

Linkage group	Mapped markers	Distinct positions	Genetic length(cM)	Marker interval(cM)
group_1	157	90	137.728	0.88
group_2	109	68	72.441	0.66
group_3	71	46	64.468	0.91
group_4	57	38	75.453	1.32
group_5	190	115	160.076	0.84
group_6	112	78	121.79	1.09
group_7	104	77	170.401	1.64
group_8	67	47	107.691	1.61
group_9	122	92	65.992	0.54
group_10	160	102	105.088	0.66
Total	1149	753	1081.128	0.94
Ge1	1090.53	–	–	–
Ge2	1101.65	–	–	–
Ge	1096.09	–	–	–
coverage	98.63%	–	–	–

**Table 5:** Summary of the linkage map for *C. hongkongensis* of SH-hybrid family

Linkage group	Mapped markers	Distinct positions	Genetic length(cM)	Marker interval(cM)
group_1	70	49	76.87	1.1
group_2	39	20	54.8	1.41
group_3	64	42	40.129	0.63
group_4	58	43	70.6	1.22
group_5	56	32	58.741	1.05
group_6	58	43	75.188	1.3
group_7	43	32	62.118	1.44
group_8	50	36	65.063	1.3
group_9	43	29	72.799	1.69
group_10	33	24	57.736	1.75
Total	514	350	634.044	1.23
Ge1	646.34	–	–	–
Ge2	660.37	–	–	–
Ge	653.36	–	–	–
coverage	97.04%	–	–	–

An average genetic map of *C. sikamea* was constructed based on 24 shared markers from HS-hybrid family 1 and SH-hybrid family 2. The average map of *C. sikamea* contained 1795 markers spanning 1346.71 cM (Table 6; Figure 3E), with marker intervals ranging from 0.44 cM in LG10 to 3.26 cM in LG11 and a mean marker interval of 0.75 cM. The expected map length was 1355.71 cM (G_e1_) and 1372.69 cM (G_e2_), with an average of 1364.20 cM, resulting in 98.72% genome coverage. Some marker intervals in the genetic maps were remarkably larger, indicating recombination hotspots.

**Table 6:** Summary of the integrated linkage map for *C. sikamea*

Linkage group	Mapped markers	Distinct positions	Genetic length(cM)	Marker interval(cM)
LG1	216	132	150.09	0.69
LG2	180	121	99	0.55
LG3	175	128	89.62	0.51
LG4	57	38	75.45	1.32
LG5	304	191	157.16	0.52
LG6	148	106	133.74	0.9
LG7	165	126	221.84	1.34
LG8	98	63	107.69	1.1
LG9	122	91	65.99	0.54
LG10	260	176	113.38	0.44
LG11	16	13	52.22	3.26
LG12	54	37	80.53	1.49
Total	1795	1222	1346.71	0.75
Ge1	1355.71	–	–	–
Ge2	1372.69	–	–	–
Ge	1364.2	–	–	–
coverage	98.72%	–	–	–

### Quantitative trait loci analysis of shell height and shell length

**Figure 4:**
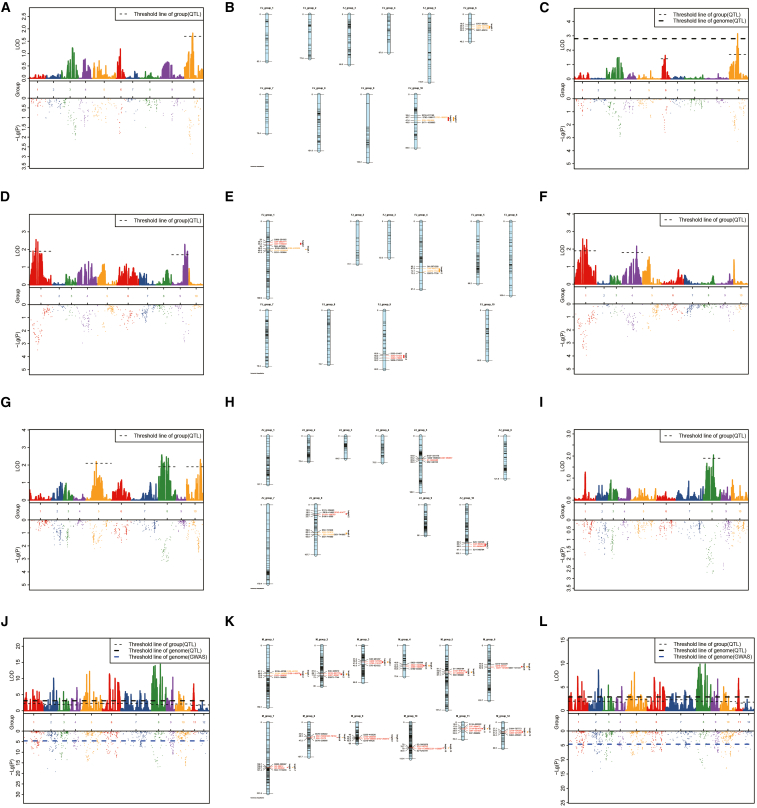
QTL mapping of shell height and shell length in the hybrid oysters (A) The QTL associated with shell height in *C. hongkongensis* of HS-hybrid family. (B) Distribution of shell height and shell length QTLs in *C. hongkongensis* of HS-hybrid family. (C) The QTLs associated with shell length in *C. hongkongensis* of HS-hybrid family. (D) The QTLs associated with shell height in *C. sikamea* of HS-hybrid family. (E) Distribution of shell height and shell length QTLs in *C. sikamea* of HS-hybrid family. (F) The QTLs associated with shell length in *C. sikamea* of HS-hybrid family. (G) The QTLs associated with shell height in *C. sikamea* of SH-hybrid family. (H) Distribution of shell height and shell length QTLs in *C. sikamea* of SH-hybrid family. (I) The QTL associated with shell length in *C. sikamea* of SH-hybrid family. (J) The QTLs associated with shell height in *C. sikamea* of combined of HS- and SH-hybrid family. (K) Distribution of shell height and shell length QTLs in *C. sikamea* of combined of HS- and SH-hybrid family. (L) The QTLs associated with shell length in *C. sikamea* of combined of HS- and SH-hybrid family.

QTL for shell height (SH) and shell length (SL) were scanned genome-wide. In the female-specific map (*C. hongkongensis*) of HS-hybrid family 1, three significant QTLs (qSH1, qSL1-1, and qSL1-2), were identified on LG10 and LG6, explaining 7.4, 6.6, and 12.4% of the phenotypic variance, respectively (Table 7; Figures 4A–C). In the male-specific map (*C. sikamea*) of HS-hybrid family 1, four significant QTLs (qSH1-1, qSH1-2, qSL1-1, and qSL1-2) were found on LG1, LG4, and LG9, explaining 10.2, 9.1, 10.2, and 8.7% of the phenotypic variance, respectively (Table 8; Figures 4D–F). In the female-specific map (*C. sikamea*) of SH-hybrid family 2, four QTLs (qSH2-1, qSH2-2, qSH2-3, and qSL2) were identified on LG5, LG8, and LG10 explaining 8.8, 10.2, 9.3, and 8.2% of the phenotypic variance, separately (Table 9; Figures 4G–I).

**Table 7:** Information of QTLs associated with shell height and shell length in *C. hongkongensis* of HS-hybrid family

Trait	QTL	Linkage group	Position/cM	LOD	Variance explained (%)
shell height	shell height −1	10	42.661	1.83	7.4
shell length	shell length −1	6	21.383	1.64	6.6
shell length	shell length −2	10	42.661	3.15	12.4

**Table 8:** Information of QTLs associated with shell height and shell length in *C. sikamea* of HS-hybrid family

Trait	QTL	Linkage group	Position/cM	LOD	Variance explained (%)
shell height	shell height −1	1	31.712	2.56	10.2
shell height	shell height −2	9	61.908	2.29	9.1
shell length	shell length −1	1	40.458	2.58	10.2
shell length	shell length −2	4	69.482	2.17	8.7

**Table 9:** Information of QTLs associated with shell height and shell length in *C. sikamea* of SH-hybrid family

Trait	QTL	Linkage group	Position/cM	LOD	Variance explained (%)
shell height	shell height −1	5	65.187	2.2	8.8
shell height	shell height −2	8	20.471	2.58	10.2
shell height	shell height −3	10	86.714	2.32	9.3
shell length	shell length −1	8	63.278	2.05	8.2

In the average map (*C. sikamea*), twelve QTLs (qSH3-1 - qSH3-12) for shell height were evenly distributed across LG1-LG12. Among them, qSH3-8 showed the largest LOD score (14.62), explaining 26.4% of the phenotypic variance, while qSH3-12 presented the smallest LOD (3.02), explaining 6.1% of the variance. Interestingly, most of the twelve QTLs (qSL3-1 – qSL3-12) for shell length were located in the same regions as those for shell height on LG1-LG12 (Table 10; Figures 4J–L).

**Table 10:** Information of QTLs associated with shell height and shell length in integrated linkage map of *C. sikamea*

Trait	QTL	Linkage group	Position/cM	LOD	Variance explained (%)
height	height.1	1	69.52	6.55	12.8
height	height.2	2	69.1	10.11	19.1
height	height.3	3	41.65	5.93	11.7
height	height.4	4	48.36	7.18	14.0
height	height.5	5	64.65	12.21	22.6
height	height.6	6	51.83	11.44	21.3
height	height.7	7	140.36	6.2	12.2
height	height.8	8	46.3	14.62	26.4
height	height.9	9	48.27	10.4	19.6
height	height.10	10	78.97	9.9	18.7
height	height.11	11	19.61	8.33	16.0
height	height.12	12	28.92	3.02	6.1
length	length.1	1	68.12	7.28	14.1
length	length.2	2	69.1	8.65	16.6
length	length.3	3	41.65	5.42	10.7
length	length.4	4	48.36	5.09	10.1
length	length.5	5	64.65	8.17	15.7
length	length.6	6	50.83	7.05	13.7
length	length.7	7	140.36	4.3	8.6
length	length.8	8	46.3	10.67	20.0
length	length.9	9	48.27	6.71	13.1
length	length.10	10	78.97	5.74	11.3
length	length.11	11	19.61	6.86	13.4
length	length.12	12	28.92	2.4	4.9

### Candidate genes associated with shell height and shell length

**Figure 5:**
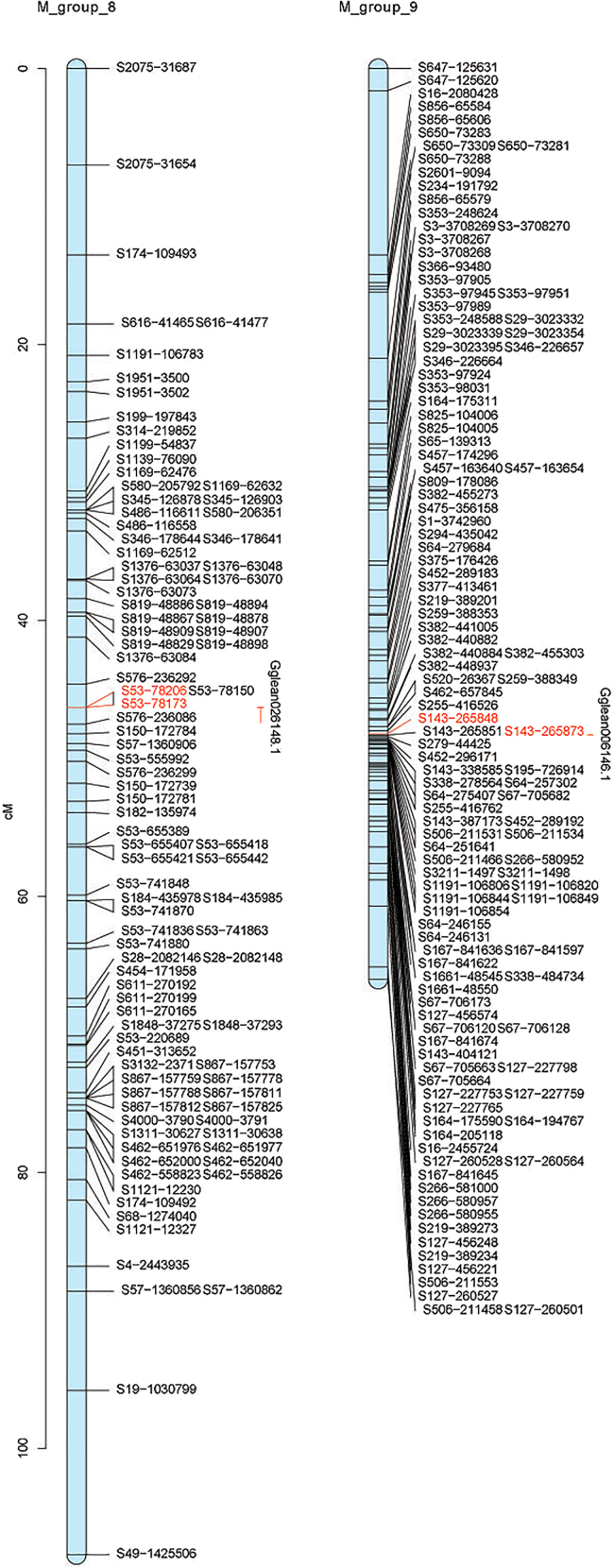
Distribution of two candidate genes for shell height and shell length in *C. sikamea* of combined of HS- and SH-hybrid family

Using the *C. hongkongensis* genome annotation,^17^ candidate genes were identified. Since the genome assembly was at the contig-level, some QTL intervals spanned two contigs, resulting in too many candidate genes. To narrow down the candidate regions, only QTL intervals where the left and right nearest markers were located on a single contig were considered. A total of 20 genes were identified in four QTL intervals, including 18 genes for SH, 20 genes for SL, based on the average genetic map of *C. sikamea* (Table 11). Among these, 18 genes were shared between SH and SL, likely due to the high correlation between the two traits. Of the 18 shared genes, two candidate genes (*Polycystin* and *Afadin*) had LOD >10 and explained more than 19% of the phenotypic variance for SH, and LOD scores >6 and explained more than 13% of the phenotypic variance for SL (Figure 5). In addition, two genes for SL were identified in the QTL interval based on the female-specific map (*C. sikamea*) of the SH-hybrid family (Table 12). Twenty-two genes from five QTL intervals were used for GO and KEGG enrichment analysis (Tables 13 and 14). Although the number of candidate genes was limited, the enrichment analysis provided insights into the genetic mechanisms underlying shell height and length traits.

**Table 11:** Summary information of candidate genes for shell height and shell length in *C. sikamea* of integrated linkage map

QTL	Linkage group	Position/cM	Marker interval	LOD	Variance explained(%)	candidate genes
height.1	1	69.52	NA(S159-140979-S151-199650)	6.55	12.8	–
height.2	2	69.1	NA(S215-501772-S307-83267)	10.11	19.1	–
height.3	3	41.65	NA(S406-127029-S783-118103)	5.93	11.7	–
height.4	4	48.36	NA(S623-182558-S495-198552)	7.18	14.0	–
height.5	5	64.65	NA(S102-879734-S1-6095264)	12.21	22.6	–
height.6	6	51.83	NA(S675-85727-S831-101094)	11.44	21.3	–
height.7	7	140.36	NA(S8-38912-S8-352934)	6.2	12.2	Major egg antigen [Crassostrea gigas]; Sodium-dependent glucose transporter 1 [Crassostrea gigas]; PREDICTED: translocating chain-associated membrane protein 1-like [Aplysia californica]; Solute carrier organic anion transporter family member 4A1 [Crassostrea gigas]; PREDICTED: uncharacterized protein LOC101235779, partial [Hydra vulgaris]; Alpha-tectorin[Crassostrea gigas]; hypothetical protein CAPTEDRAFT_228244 [Capitella teleta]; hypothetical protein CGI_10016017 [Crassostrea gigas]; Acetolactate synthase-like protein[Crassostrea gigas]; viral A-type inclusion protein [Trichomonas vaginalis G3]; Solute carrier organic anion transporter family member 4A1[Crassostrea gigas]; E3 ubiquitin-protein ligase UBR5 [Crassostrea gigas]; hypothetical protein CGI_10001408 [Crassostrea gigas]; Latent-transforming growth factor beta-binding protein 4 [Crassostrea gigas]; kinase C-binding protein 1 [Crassostrea gigas]
height.8	8	46.3	S53-78150(S53-78206-S53-78173)	14.62	26.4	Polycystin-2 [*Crassostrea gigas*]
height.9	9	48.27	S143-265851(S143-265848-S143-265873)	10.4	19.6	Afadin [*Crassostrea gigas*]
height.10	10	78.97	S33-1138892(S23-142702-S33-1138897)	9.9	18.7	–
height.11	11	19.61	S119-651475(S37-554472-S99-1221601)	8.33	16.0	–
height.12	12	28.92	NA(S219-724300-S382-227298)	3.02	6.1	–
length.1	1	68.12	NA(S159-46765-S159-140958)	7.28	14.1	CAS1 domain-containing protein 1 [Crassostrea gigas]; PREDICTED: E3 ubiquitin-protein ligase HERC2-like [Aplysia californica]
length.2	2	69.1	NA(S215-501772-S307-83267)	8.65	16.6	–
length.3	3	41.65	NA(S406-127029-S783-118103)	5.42	10.7	–
length.4	4	48.36	NA(S623-182558-S495-198552)	5.09	10.1	–
length.5	5	64.65	NA(S102-879734-S1-6095264)	8.17	15.7	–
length.6	6	50.83	NA(S675-85727-S831-101094)	7.05	13.7	–
length.7	7	140.36	NA(S8-38912-S8-352934)	4.3	8.6	同上
length.8	8	46.3	S53-78150(S53-78206-S53-78173)	10.67	20.0	Polycystin-2 [*Crassostrea gigas*]
length.9	9	48.27	S143-265851(S143-265848-S143-265873)	6.71	13.1	Afadin [*Crassostrea gigas*]
length.10	10	78.97	S33-1138892(S23-142702-S33-1138897)	5.74	11.3	–
length.11	11	19.61	S119-651475(S37-554472-S99-1221601)	6.86	13.4	–
length.12	12	28.92	NA(S219-724300-S382-227298)	2.4	4.9	–

**Table 12:** Summary information of candidate genes for shell length in *C. sikamea* of SH-hybrid family

QTL	Linkage group	Position/cM	Marker interval	LOD	Variance explained(%)	candidate genes
height.1	5	65.187	NA(S440-350651-S1-5000495)	2.2	8.8	–
height.2	8	20.471	NA(S616-41477-S1191-106783)	2.58	10.2	–
height.3	10	86.714	NA(S33-993637-S2-3606400)	2.32	9.3	–
length 1	8	63.278	NA(S53-741870-S53-741836)	2.05	8.2	Corticotropin-releasing factor-binding protein [Crassostrea gigas]; LDLR chaperone MESD [Crassostrea gigas]

**Table 13:** Gene Ontology enrichment analysis

id	term	Category	ListHits	ListTotal	PopHits	PopTotal	pval	padj	Enrichment_score	Gene
GO:0030976	thiamine pyrophosphate binding	molecular_function	1	6	1	6357	0.00E+00	0.00E+00	1059.50	Gglean032303.1
GO:0019222	regulation of metabolic process	biological_process	1	6	4	6357	4.45E-06	2.00E-05	264.88	Gglean032299.1
GO:0000287	magnesium ion binding	molecular_function	1	6	23	6357	1.86E-04	5.59E-04	46.07	Gglean032303.1
GO:0009792	embryo development ending in birth or egg hatching	biological_process	1	6	69	6357	1.69E-03	3.81E-03	15.36	Gglean032303.1
GO:0005488	binding	molecular_function	2	6	438	6357	5.55E-03	9.52E-03	4.84	Gglean032306.1; Gglean007557.1
GO:0008270	zinc ion binding	molecular_function	1	6	135	6357	6.35E-03	9.52E-03	7.85	Gglean032304.1
GO:0008152	metabolic process	biological_process	1	6	236	6357	1.86E-02	2.40E-02	4.49	Gglean032303.1
GO:0003824	catalytic activity	molecular_function	1	6	411	6357	5.26E-02	5.33E-02	2.58	Gglean032303.1
GO:0016020	membrane	cellular_component	1	6	414	6357	5.33E-02	5.33E-02	2.56	Gglean032298.1

**Table 14:** KEGG enrichment analysis

id	term	ListHits	ListTotal	PopHits	PopTotal	pval	padj	Enrichment_score	Gene
ko04120	Ubiquitin mediated proteolysis	2	4	173	5522	1.18E-04	9.45E-04	15.96	Gglean007557.1; Gglean032306.1
ko04670	Leukocyte transendothelial migration	1	4	70	5522	9.35E-04	3.74E-03	19.72	Gglean006146.1
ko04530	Tight junction	1	4	98	5522	1.83E-03	4.68E-03	14.09	Gglean006146.1
ko04520	Adherens junction	1	4	111	5522	2.34E-03	4.68E-03	12.44	Gglean006146.1
ko04141	Protein processing in endoplasmic reticulum	1	4	147	5522	4.08E-03	6.52E-03	9.39	Gglean032298.1
ko04014	Ras signaling pathway	1	4	184	5522	6.34E-03	8.45E-03	7.50	Gglean006146.1
ko04015	Rap1 signaling pathway	1	4	217	5522	8.75E-03	9.97E-03	6.36	Gglean006146.1
ko04024	cAMP signaling pathway	1	4	232	5522	9.97E-03	9.97E-03	5.95	Gglean006146.1

### Characterization and functional analysis of the *polycystin* gene

**Figure 6:**
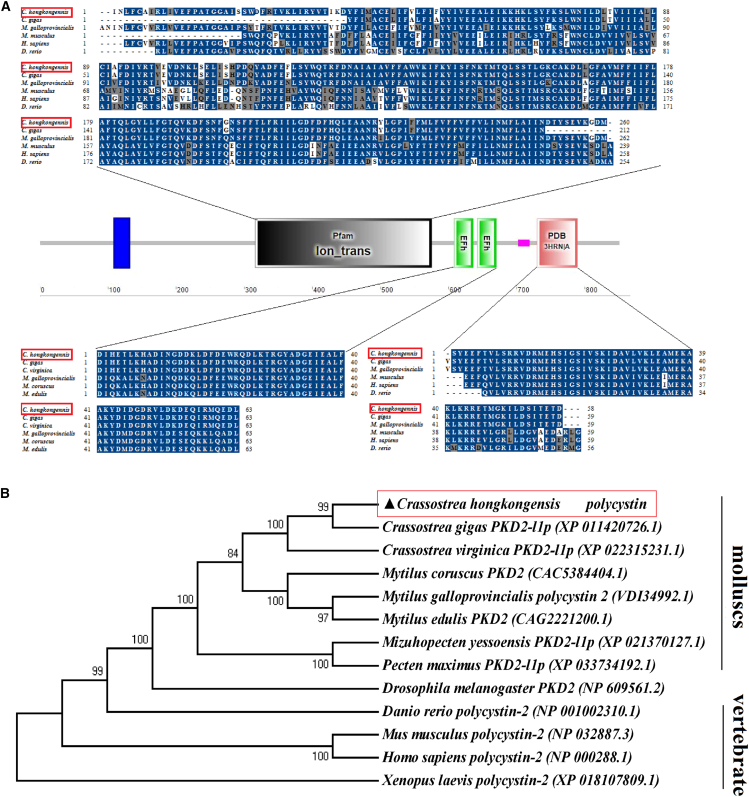
Sequence analysis of *Ch-polycystin* (A) Sequence alignment results of conservative structural domains of *Ch-polycystin* with highly homologous proteins in other species, The blue shaded sequences indicate residues that exactly match the consensus. The gray shaded sequences show a similar relationship. The protein sequences used for alignment analysis were as follows: *Crassostrea gigas* PKD2 (XP_011420726.1); *Crassostrea virginica* PKD2 (XP_022315231.1); *Mytilus galloprovincialis* polycystin-2 (VDI34992.1); *Mytilus coruscus* PKD2 (CAC5384404.1); *Mytilus edulis* PKD2 (CAG2221200.1); *Mus musculus* polycystin-2 (NP_032887.3); *Homo sapiens* polycystin-2 (NP_000288.1); *Danio rerio* polycystin-2 (NP_001002310.1). (B) A phylogenetic tree showing the relationships among *Ch-polycystin* and polycystin from different species was constructed by the neighbor-joining method using MEGA 6.0 software. The node values represent the percent bootstrap confidence derived from 1000 replicates. The GenBank accession numbers corresponding to the *Ch-polycystin* sequences and polycystin sequence examined as shown.

*Polycystin* was chosen for further study due to its highest LOD score and contribution to phenotypic variance. The full-length cDNA sequence of *Polycystin* was 3071 bp, with an ORF of 2556 bp, a 313 bp 3′-untranslated sequence, a 202 bp 5′-untranslated sequence, an ATG and TAG in-frame start and stop codon, respectively (GenBank accession: OP554213). The ORF of *Polycystin* encoded a deduced protein of 852 amino acids. Conserved domain analysis using SMART revealed that *Polycystin* protein possesses a transmembrane domain, an Ion_trans domain, two EFh domains, a low complexity domain, and a 3HRN/A domain (Figure 6A). Multiple sequence alignment showed high sequence identity with mollusks’ *polycystin-2* homologs, such as 97% identity with *C. gigas*, and 78% with *Mytilus galloprovincialis*. Besides, the sequence identity of *Polycystin* shared 52%, 50%, and 51.99% similarity with *Homo sapiens*, *Mus musculus*, and *Danio rerio*, respectively. Phylogenetic analysis clustered *Polycystin* with molluscan polycystin-2 homologs, followed by homologs from *Drosophila melanogaster*, *D. rerio*, *M. musculus*, *H. sapiens*, and *Xenopus laevis* (Figure 6B).

**Figure 7:**
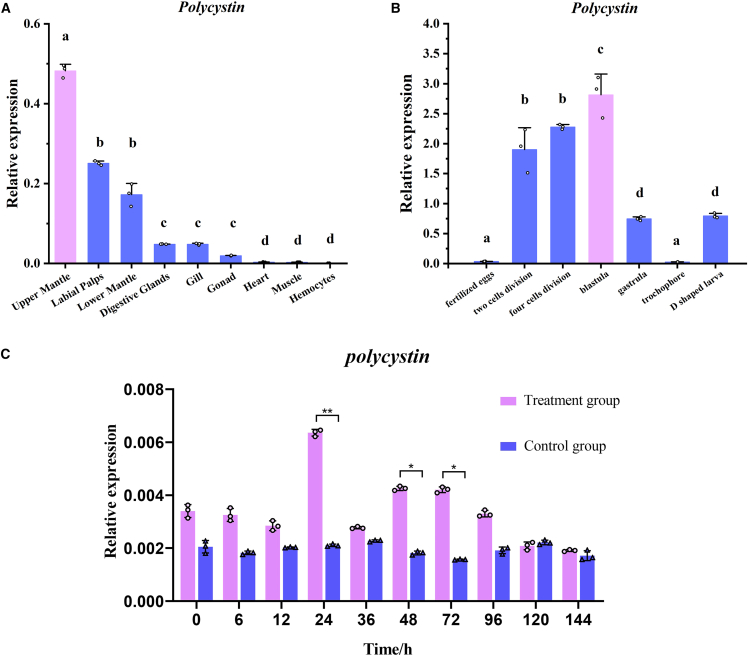
Expression analysis of *polycystin* by qRT-PCR in *C. hongkongensis* (A) The tissue distribution of *polycystin* in different tissues, where the GAPDH gene was used as a reference gene. (B) The expression profile of *polycystin* in early developmental stages, where the GAPDH gene was used as a reference gene. (C) The temporal expression of *polycystin* after shell notching in the mantle, where the GAPDH gene was used as a reference gene. Note: The significance of data was analyzed using one-way ANOVA (for A and B) and *t* test (for C) by the SPSS software. The dots represent biological replicates, and the vertical bars represent the mean ± SD (*N* = 3). In the same graph, different letters indicate significant differences (*p* < 0.05). ∗ and ∗∗ indicate a significant difference at *p* < 0.05 and *p* < 0.01, respectively, at the same point in time.

To study the spatial distribution of *Polycystin* in two oyster species, tissue-specific and developmental stage-specific mRNA expression levels of *Polycystin* were examined using q-PCR technology. The results revealed that the expressions of *Polycystin* on two oyster species showed a gradual increase from the fertilized egg stage to the blastula stage, peaking at the blastula stage (*p < 0.05*) (Figure 7B; Figure S1B). In spatial distributions of *Polycystin* gene, the expressions of *Polycystin* gene in the upper mantle were the highest among all tissues on two oyster species (*p < 0.05*) (Figure 7A; Figure S1A). In the shell notching experiment, *Polycystin* expression reached its highest level at 24 h in *C. hongkongensis* and 48 h in *C. sikamea* (*p < 0.01*), followed by a decline and subsequent upregulation at 48 h or 120 h, respectively (Figure 7C; Figure S1C).

**Figure 8:**
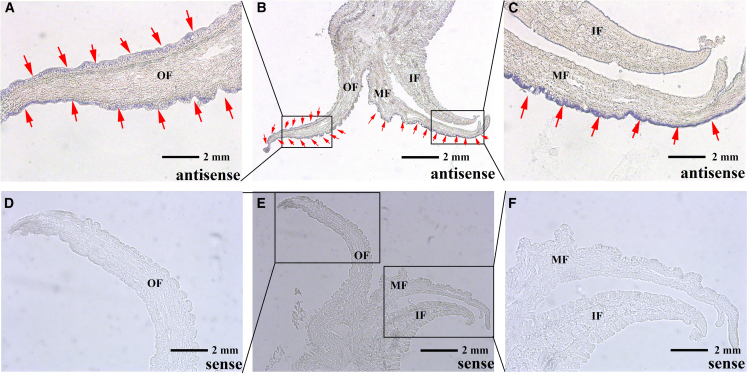
*In situ* hybridization of *polycystin* in mantle tissue of *C. hongkongensis* The red arrows indicate the positive signal. A positive signal is indicated by the blue color. IF represents inner fold, MF represents middle fold, OF represents outer fold. (A–C) The picture of the experimental group. The red arrows are indicated the positive signal. (D–F) The picture of the control group. Bars = 2 mm.

The locations of *Polycystin* mRNA in the mantle of adult *C. hongkongensis* and *C. sikamea* were confirmed by the result of ISH. *Polycystin* mRNA was detected at the corner and middle folds (Figures 8A–C; Figures S2A–C), with no signal in the central zone or inner fold (Figures 8D–F; Figures S2D–F).

**Figure 9:**
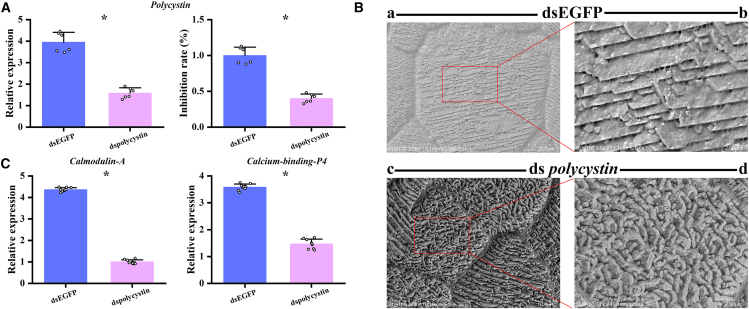
RNA interference of *polycystin* in *C. hongkongensis* (A) The expression of *polycystin* was determined 6 days after 100 μg dsRNA injection by q-PCR, where GAPDH gene was used as a reference gene. (B) SEM images of prismatic layer after injecting dsRNA. (a, b) 2.00 k× and 20.00 k×SEM images of the prismatic layer of dsEGFP group, respectively; (c, d) 2.00 k× and 20.00 k× SEM image of the prismatic layer of ds*Ch-polycystin* group, respectively. In a and c, bars = 10.0 μm. In b and d, bars = 2.00 μm. (C) Relative expression levels of Calmodulin-A, Calcium-binding-P4 after RNAi, where GAPDH gene was used as a reference gene. Note: The vertical bars represent the mean ± SD (*N* = 3, biological replicates). The significance of data was analyzed using *t* test by the SPSS software. ∗ indicates a significant difference at *p* < 0.05 between the ds EGFP groups and ds *polycystin* groups.

To functionally characterize *Polycystin* in the shell formation of *C. hongkongensis* and *C. sikamea*, RNAi technology was utilized. The results (Figure 9A; Figure S3A) revealed that the RNAi efficiency was 62.4% and 63.9% in *C. hongkongensis* and *C. sikamea*, respectively. SEM results revealed that the control group (dsEGFP injected) showed a normal prismatic structure with clear, well-defined nodular growth and orderly arrangement of calcite mineral layers, while the experimental group (ds*Polycystin* Injected) showed irregular mineral deposition (Figure 9B; Figure S3B). qPCR analysis of mineralization-related genes revealed that *Polycystin* knockdown significantly downregulated other calcium-regulated genes (e.g., *Calmodulin-A* and *Calcium-binding-P4*), suggesting synergistic roles in shell formation (Figure 9C; Figure S3C).

## Discussion

In oyster studies, many efforts have focused on the evaluation of oysters’ hybridization.^5^^,^^19^ Identifying the genetic bases of genomic incompatibility and heterosis is vital to promote the development of oyster breeding and genetic improvement technologies. In this study, interspecific hybrid families were first used to construct genetic maps. Thanks to the modified GBS method, high-resolution genetic linkage maps of *C. hongkongensis* and *C. sikamea* were constructed through cost-effective genotyping of reciprocal hybrid families of 220 oysters. The mapping density of *C. hongkongensis* was 1.5 cM with 97.30% coverage in family 1 and 1.23 cM with 97.04% coverage in family 2. A consensus map was not constructed due to insufficient shared markers. The mapping density for *C. sikamea* was 1.22 cM with 97.40% coverage in family 1 and 0.94 cM with 98.63% coverage in family 2. A consensus map was constructed based on the 24 shared markers, with a mapping density of 0.75 cM and 98.72% coverage. This study provides the first linkage maps for *C. hongkongensis* and *C. sikamea*. Compared to the published linkage maps of the interspecific hybrid resource family with first generation markers,^20^ the genetic maps constructed in this study have higher density and more markers. Recently, Mao et al.^21^ constructed high density second-generation linkage maps for interspecific hybrids of two scallops, with mean marker spacing of 0.32 cM and 0.51 cM using the 2b-RAD method. These maps offered a substantial improvement over first generation maps. Major differences in polymorphic sites were identified between the two scallop species, with all mapping markers being species-specific polymorphic sites. Mao et al.^21^ also observed that about 60% of the markers were species-specific polymorphic between the two scallop species. In the current linkage map, a few marker intervals (i.e., 42.5 cM in LG9, family 1) were remarkably large, indicating hotspots of recombination. Thus, additional markers from these regions are needed to further improve the genetic linkage map. In addition, only 1.5% (family 1) and 0.9% (family 2) shared codominant markers were identified between the two species, causing a lack of sufficient markers for the construction of consensus maps.

Female maps have been reported to be longer than male maps,^22^^,^^23^ and similar observations have been documented in oysters.^13^^,^^24^ However, Hedgecock et al.^25^ documented that the recombination rates between male and female were statistically insignificant, suggesting that sex-specific recombination patterns are highly dependent on the mapping families. In the current study, the female-to-male recombination ratio in family 2 was 1.70:1, which was in agreement with the results of previous studies. However, the female-to-male recombination ratio in family 1 was 1.07:1 (*p* < 0.05), supporting the idea that sex-specific recombination patterns are highly dependent on the mapping families. The recombination of chromosomes in males and females often differs regionally, with higher recombination in regions proximal to centromeres and telomeres in females and males, respectively.^26^ In this study, the linkage group length showed an obvious inconsistency between female and male maps in family 1 and family 2. For instance, LG7 was the largest and smallest LG on the female and male maps, respectively. LG10 had the most mapped markers on the maternal map, while LG10 was fairly small, and LG7 had the most mapped markers on the paternal map in family 1. A similar observation has been documented in hybridized families of *C*. *gigas* and *C. angulate*, in which at least one pair of corresponding LGs with different lengths has been identified between female and male maps.^13^

Segregation distortion is common in oysters, and the degree of distorted markers varies significantly across different crosses.^25^^,^^27^ In this study, we observed 57.8% and 60.8% distorted markers in reciprocal F1 hybrid families, which is comparable to the results of other studies.^13^^,^^21^ Hybrid incompatibility is an important factor responsible for the uneven transmission of alternate alleles.^28^ Since distorted markers contribute to the increase in genetic map coverage and linked QTL detection, their deletion is expected to cause massive information loss and a decrease in genome coverage.^29^ Therefore, these distorted markers were retained for map construction.

QTLs for growth-related traits have been identified in *C. gigas*.^13^^,^^27^ As revealed by Zhang et al.,^5^ the hybrids of *C. hongkongensis* and *C. sikamea* exhibited higher growth heterosis and survival advantage. The oyster shell shape in the adult stage is highly affected by the environment; the measured data of shell height and shell length are not always accurate. In order to improve the accuracy of QTL mapping, we selected hybrid families aged 4 months for measurement. In the present study, shell growth-related traits were highly correlated with growth-related QTLs, suggesting that shell growth-related traits and growth-related traits might be regulated by the same set of QTLs. This speculation was confirmed, as most QTLs correlated with shell length were found in the same region corresponding to shell height. A total of 35 significant QTLs for shell growth-related traits were detected in the two oyster species. Among them, the variance explained by these QTLs ranged from 6.6% to 12.4% based on LGs of family 1 and family 2, respectively, which is comparable to *C. gigas*,^27^ but much lower than that of golden pompano (16.6–21.3%).^30^ However, the variance explained by most QTLs ranged from 10.1% to 26.4% based on LGs of the average map (*C. sikamea*), which is significantly higher than previous QTLs. The cause of such differences could be partly explained by the resolution of the genetic maps.^23^^,^^31^ In this study, 20 genes within the QTL intervals were identified to be involved in both physiological processes and biochemical reactions, such as TECTA, UBR5, LTBP4, and Afadin. Alpha-tectorin (TECTA) is a TH-responsive gene, encoding the major non-collagenous glycoproteins and the tectorial membrane.^32^^,^^33^ UBR5 is an E3 ubiquitin ligase that plays an important role in recovery, hypertrophy, and anabolism from atrophy in skeletal muscle.^34^ LTBPs play an important role in the secretion and folding of transforming growth factor-βs (TGF-βs), which monitor the growth and differentiation of various cell types, as well as the homeostasis of extracellular proteolysis.^35^ Afadin is an actin-binding protein that plays an important role in the formation of adberens junctions.^36^ Other genes identified in QTL regions, such as SGLT and SLCO4A, are known to be involved in transporters, and PRKCBP1 is known to be involved in signal transduction. Moreover, we also screened a few candidate genes with unknown functions. In particular, the *Polycystin* gene, identified within the QTL with the highest LOD and phenotypic variation, captured our interest.

The analysis of the amino acid functional domain of *Polycystin* showed that it contains a transmembrane domain, an Ion_trans domain, two EFh domains, a low complexity domain, and a transport protein domain (PDB id: 3HRN/A). *Polycystin* possesses a transmembrane domain of 12 amino acids in length at the front end and a typical ion channel domain (Ion_trans), indicating that this protein is a transmembrane protein and may function as an ion channel protein. Generally, ion channel proteins usually contain six transmembrane helices, the last two of which are flanked by a loop that determines the ion selectivity.^37^ *Polycystin* has two EFh domains, demonstrating that *Polycystin* encodes a calcium channel protein. The EFh domain is a calcium binding motif that occurs at least in pairs and undergoes conformational changes upon binding to calcium ions.^38^ Furthermore, low-complexity domains have been reported to play a role in the aqueous phase separation of calcium ions and the binding of related proteins.^39^ The 3HRN/A domain is a helix-like domain at the C-terminus of the TRPP2 protein family and is essential for the formation of ion channel complexes.^40^ Multiple sequence alignment and phylogenetic analysis showed that *Polycystin* is highly conserved in the Ion_trans domain, EFh domains, and 3HRN/A domain, and is relatively evolutionarily conserved in shellfish. All these results indicate that *Polycystin* may have been functionally conserved across various evolutionary species.

Many studies have confirmed that the mantle is the main tissue for calcium turnover and deposition in molluscan.^41^^,^^42^^,^^43^ In addition, abundant mineralization-related proteins have been reported in the mantle, and *in vitro* studies have confirmed their mineralization functions.^44^^,^^45^ In adult oysters, the q-PCR expression of *Polycystin* was highest in the upper mantle of two oysters (*p < 0.05*), preliminarily indicating that *Polycystin* may be associated with shell formation in oysters. The expression profile of *Polycystin* during oyster developmental stages all showed a gradual increase from the fertilized egg stage to the blastula stage, with the highest expression at the blastula stage (*p < 0.05*). This has also been confirmed in other studies, as early shell formation begins at the non-shelled trochophore larvae stage in bivalves.^46^^,^^47^ Thus, the period prior to the trochophore can be seen as a preparatory stage for early shell formation, which includes processes such as calcium ion recruitment, matrix protein synthesis, and secretion.^42^^,^^47^^,^^48^ Above all, we speculate that *Polycystin* might be related to calcium ion recruitment prior to the early shell formation to provide an ionic environment with sufficient calcium ions for the shell formation process during the larvae stage.

We also studied the expression profile of *Polycystin* during the shell repair process to gain insight into its potential role in the shell formation process of oysters. The results indicated that *Polycystin* functioned as a positive regulator during shell regeneration and formation. Mount et al.^49^ also studied shell formation in adult oysters by notching the shell margin and revealed that notching triggered the activation of shell formation-related genes, with the prismatic layers regenerated in the notched region. Similar results have been found in biomineralization studies of other shellfish. In *Chlamys farreri*, shell notching caused significant up-regulation of *calponin* expression after 6 h.^50^ In *Pinctada fucata* after 24 h of shell notching, the expression of *PfChi1* increased significantly.^51^ In this study, *Polycystin* gene expression was upregulated and peaked at 24 h or 48 h in two species, respectively, thus suggesting that *Polycystin* might play an important role in the early stage of shell regeneration. The long term relatively high expression of *Polycystin* might indicate that it also plays an important role in shell growth.

Shell formation in shellfish is an extracellular process controlled by the organic matrix secreted by the mantle epithelium.^42^^,^^44^ In bivalves, the mantle is usually divided into three parts: the mantle edge (ME), mantle center (MC) and mantle pallial (MP). The mantle edge (ME) has 3-folds: the outer fold (OF), middle fold (MF), and inner fold (IF), each performing distinctive functions.^50^^,^^52^^,^^53^ The results of *in situ* hybridization revealed that *Polycystin* was expressed on the edge of both the OF and MF. The mantle epithelial cells are capable of absorbing inorganic ions from the external aqueous environment into the body through active transport and later translocating them into the mantle cavity to form a highly concentrated ionic environment.^42^^,^^54^^,^^55^ In addition, the OF is the closest mantle fold structure to the external water environment, and the epithelial cells distributed at the edge of the OF also perform the function of sensing the external water environment, and the OF was the sole effective tissue among the three mantle folds with regard to oyster shell color.^41^^,^^55^ The ions are then transported to the mantle cavity, where liquid precursors are transformed into solid biominerals through bio-mineralization.^56^^,^^57^ Combined with the previous results of the predicted protein functional domain of *Polycystin*, we speculate that the protein encoded by *Polycystin* likely functions as a calcium channel protein distributed at the edge of the outer and middle folds, mediating the entry of calcium ions from the external aqueous environment into the mantle cavity. It has been suggested that the area between the OF and MF is called the periostraca groove and is mainly responsible for the secretion of proteins required for cuticle formation.^55^^,^^58^ In addition, it has also been pointed out that the mantle edge is mainly responsible for prismatic layer formation.^42^^,^^59^^,^^60^ The *in situ* hybridization results did not give a clear indication of whether *Polycystin* is involved in cuticle or prismatic formation. Therefore, shell notching and RNA interference experiments were conducted to further investigate the exact role of *Polycystin* in the shell formation of oysters.

The results of RNA interference revealed that silencing *Polycystin* disrupted shell biomineralization. We found that in the control groups, uniform mineralized crystals with clear edges were observed. In contrast, the experimental group (ds*Polycystin* Injected) showed irregular deposition of mineral layers. A similar observation has been reported in *Chlamys farreri*, in which the interference of *calponin* expression caused irregular growth of the shell crystals.^50^ Moreover, knocking down the *PfmPif97-like* gene also caused fragmentation in the prismatic layer of *Pinctada fucata martensii*.^61^ The SMART analysis of *Polycystin* showed that it has the typical characteristics of the EFh superfamily, which is shared by calmodulin (CaM) and calmodulin-like protein (CaLP). It has been demonstrated that CaM and CaLP were involved in the calcium metabolic pathway and play a regulatory role in the growth of the prismatic layer in *P. fucata*.^62^^,^^63^^,^^64^^,^^65^ In mice, it was found that knockdown of PKD1, PKD2, and Kif3a by gene editing in osteoblasts and osteoclasts leads to the reduced formation of new bone and also to downregulate the expression of osteogenic-related genes in the bone, such as osteocalcin and osteosalivary protein.^66^^,^^67^^,^^68^ Therefore, we speculate that *Polycystin* has two main functions in the shell formation of oysters: on the one hand, it acts as a Ca^2+^ channel protein to transport sufficient calcium ions into the mantle cavity to complete the pre-mineralization phase, and on the other hand, it may indirectly regulate the orderly deposition of calcium carbonate crystals by participating in calcium signaling pathways. After interfering with the expression of *Polycystin* by RNAi, we found that the relative expressions of *Calmodulin-A* and *Calcium-binding-P4* were also downregulated. It has been confirmed that *Calmodulin-A* and *Calcium-binding-P4* are associated with the transport of Ca^2+^ and play important roles in the biomineralization process.^55^^,^^63^^,^^69^ Therefore, we speculate that *Polycystin* might indirectly regulate the shell crystal structure by regulating the expression of other mineralization-related genes. This study provides evidence of the biomineralization function of *Polycystin* in mollusks.

### Limitations of the study

In this study, GBS methods were employed for genetic linkage map construction, which might affect the number of markers mapped. Subsequent research could utilize whole-genome resequencing for map construction. Additionally, as the reference genome used was at the contig-level, candidate genes within QTL regions might have been overlooked. Further analysis could be conducted using a chromosome-level reference genome for more accurate results. Our study mainly used high-resolution genetic linkage maps, QTL mapping, and related functional studies to confirm that the *Polycystin* gene indeed participated in the shell formation of *Crassostrea hongkongensis* and *C. sikamea*, but these findings may not be applicable to unstudied *Crassostrea* species or other oyster populations. Based on all results, we speculate that *Polycystin* has two main functions in the shell formation: on the one hand, it acts as a Ca^2+^ channel protein to transport sufficient calcium ions into the mantle cavity to complete the pre-mineralization phase, and on the other hand, it may indirectly regulate the orderly deposition of calcium carbonate crystals by participating in calcium signaling pathways. However, further experimentation will be needed to confirm the above functional hypotheses regarding the *Polycystin* gene.

## Resource availability

### Lead contact

Further information and requests for resources and reagents should be directed to and will be fulfilled by the lead contact, Yuehuan Zhang (yhzhang@scsio.ac.cn).

### Materials availability

Materials produced in this study are available from the lead contact on a collaborative basis.

### Data and code availability

• GBS sequencing data is deposited in NCBI under the accession number of NCBI-SRA (PRJNA967088), see also in key resources table.
• The script which transformed the vcf format to JoinMap format is deposited in https://github.com/JiaoBingke/LinkageMapAndQTL/tree/main, see also in the key resources table.
• Any additional information required to reanalyze the data reported in this article is available from the lead contact upon request.

## Acknowledgments

This research was supported by the 10.13039/501100021171Guangdong Basic and Applied Basic Research Foundation (2023A1515010944; 2021A1515011181; 2022A1515010203); the Hainan Provincial Key R&D Programme (ZDYF2024XDNY175; ZDYF2021XDNY135; ZDYF2021XDNY183); Guangzhou Science and Technology Project (202206010133); the 10.13039/501100001809National Science Foundation of China (31872566; 31702340; 32002387); the Guangdong Provincial Key Research and Development Program (2021B0202020003); the Project of Sanya Yazhou Bay Science and Technology City; the Science and Technology Project of Guangdong Provincial Department of Natural Resources (GDNRC[2022]40); the Innovation-driven Development Project of Guangxi, China (AA19254032); the National Marine Genetic Resource Center; the earmarked fund for CARS-49; the Science and Technology Planning Project of Guangdong Province, China (2023B1212060047); and the Research on breeding technology of candidate species for the Guangdong modern marine ranching (2024-MRB-00-001). We thank Rongchao Zhang, a bioinformatics engineer at Qingdao OE Biotech Ltd., for his guidance on the bioinformatics analysis.

## Author contributions

Y.Z. and Z.Y. conceived the study. H.M. and Y.Q. carried out the field and laboratory work, and drafted the article. D.Y. and B.J. participated in the data analysis. Q.L. and J.L. conducted phenotypic characterizations. Q.L. and Y.Z. collected the samples. Y.Z. and J.H. carried out the bioinformatic analyses. All authors evaluated the results and revised the article.

## Declaration of interests

The authors declare no competing interests.

## STAR★Methods

### Key resources table

**Table:** 

REAGENT or RESOURCE	SOURCE	IDENTIFIER
**Critical commercial assays**		

SMARTer® RACE 5’/3′ Kit	TAKARA, Kusatsu, Japan	Cat#634499
PrimeScriptⅡ1st Strand cDNA Synthesis Kit	TAKARA, Kusatsu, Japan	Cat#6210B
T7 RiboMAX™ Express RNAi System	Promega, USA	Cat#P1700
DIG RNA Labeling Kit (SP6/T7)	Roche, Basel, Switzerland	Cat#11175025910
pMD19-T Vector Cloning Kit	TAKARA, Kusatsu, Japan	Cat#6013
ClonExpress II One Step Cloning Kit	Vazyme, China	Cat#C112-01
2×RealStar Green Power Mixture	Genstar, China	Cat#A314-10
Taq master mix	NEB	Cat#M0270L

**Deposited data**		

GBS reads	This paper	NCBI-SRA (PRJNA967088)

**Experimental models: Organisms/strains**		

*C. hongkongensis*	Zhuhai, Guangdong Province, China	N/A
*C. sikamea*	Zhanjiang, Guangdong Province, China	N/A

**Oligonucleotides**		

Primers for clone of *Polycystin* gene and RNA FISH etc, see Table S1	This paper	N/A

**Recombinant DNA**		

pEGFP-N1	Solarbio, Beijing, China	Cat#P6460

**Software and algorithms**		

bowtie 2	Langmead and Salzberg^70^	version 2.2.3.4.3
GATK	DePristo et al.^71^; Van der Auwera et al.^72^	version 3.8
Vcftools	Danecek et al.^73^	version 0.1.16
JoinMap	Van Ooijen^74^	version 4.0
MergeMap	Wu et al.^75^	https://github.com/ucrbioinfo/MergeMap?tab=readme-ov-file
MapQTL 6.0	Van Ooijen.^76^	version 6.0
R Statistical Software	R Foundation for Statistical Computing	R 4.2.3
SPSS	SPSS Inc., USA	verision 19.0
ORF Finder	NCBI, USA	https://www.ncbi.nlm.nih.gov/orffinder/
Simple Modular Architecture Research Tool	NCBI, USA	https://smart.embl.de/smart/change_mode.cgi
Clustal W	Larkin et al.^77^	verision 2.0
MEGA	Tamura et al.^78^	verision 11.0
vcf2joinmap_x2.F1.pl	This paper	https://github.com/JiaoBingke/LinkageMapAndQTL

### Experimental model and study participant details

Sexually mature wild *C. hongkongensis* were collected from Zhuhai, and the 2nd generation of *C. sikamea* broodstock were collected from Zhanjian. Among them, the wild *C. hongkongensis* were collected on May 10, 2018, from the waters near Hengqin Island in Zhuhai, with the sampling area having a water depth of approximately 10 m, a water temperature of 28.5°C, and a salinity of 20‰. All oysters were maintained at the Zhanjiang Marine Economic Animal Station hatchery of the Chinese Academy of Sciences. Species identification was based on gill tube structure and shell morphology, following the methods of Wang et al.^79^

### Method details

#### Mapping families and trait measurement

Two mapping families, HS-hybrid family 1 and SH-hybrid family 2, were established by crossing *C. sikamea* and *C.hongkongensis*. HS-hybrid family 1 was produced by crossing a *C. hongkongensis* female with a *C. sikamea* male, while SH-hybrid family 2 was produced by crossing a *C. hongkongensis* male with a *C. sikamea* female. The mapping families consisted of 220 individuals, 110 from HS-hybrid family 1 and 110 from SH-hybrid family 2. Hybrid family breeding, from fertilization to grow-out, was performed according to Zhang et al.^5^ At four months age, the shell height and shell length of the 220 progenies of HS-hybrid family 1 and SH-hybrid family 2 were measured and recorded. Soft tissue from each oyster were extracted, preserved in 70% ethanol, and stored at −80°C.

#### Genetic confirmation of hybrid progeny

Two hundred and twenty progenies from HS-hybrid family 1 and SH-hybrid family 2 were examined at four months of age. Genomic DNA was extracted according to the standard phenol-chloroform protocol.^80^ Species identification of the reciprocal hybrid progeny was performed using the SSR marker *Ch409*. The primer sequences were 5′-CGACTGGTGGGAGTTTCTGAC-3’ (forward) and 5′- GCCGCTTCTATCTCCTTTGC-3’ (reverse). PCR was performed in 25 μL volumes containing 2.0 mM MgCl_2_, 0.2 mM dNTP, 0.2 mM of each primer, 50 ng of template DNA, 1U Taq polymerase and 2.5 μL of 10 × PCR buffer. The PCR conditions included an initial denaturation at 94 °C for 5 min; 30 cycles of 94 °C for 30 s, 60 °C for 30 s, and 72 °C for 1 min; and a final extension at 72°C for 10 min. Three controls (DNA from a *C. sikamea* parent, DNA from a *C. hongkongensis* parent, and DNA from reciprocal hybrid progeny) were also included. Amplified fragments were separated by gel electrophoresis, with a 50-bp DNA ladder (Takara) used as reference markers.

#### GBS library preparation and sequencing

Libraries were prepared using modified GBS methods according to the method of Qi et al.^81^ A total of 200 ng genomic DNA from each individual was digested in a 30 μl reaction using methylation sensitive restriction enzymes 8U *MspI* and 8U *PstI* HF (NEB) at 37 °C for 2 h. The restriction enzymes were inactivated by incubation at 75 °C for 20 min. A total of 20 μl of the restriction digest was mixed with 1.5 μl of common *MspI* adapater, 1 μl of barcoded *PstI* HF adapter (stock: 0.1μM), 200U of T4 DNA ligase (NEB) and 4 μl of 10 × T4 DNA ligase buffer. Ligation was performed at 22 °C for 2 h, fragments < 300bp were eliminated using Sera-Mag Speed Beads (GE Healthcare Life Sciences) (0.7 volumes) at room temperature for 5 min. A magnetic stand was used to separated beads from the supernatant, and then washed three times with 200 μl of 70% ethanol. DNA was eluted from the air-dried beads using 40 μl of 10 mM Tris-HCl (pH 8.0). A 3 μl of the eluate was transferred to a cocktail containing 5 μl of 5 × Taq master mix (NEB), 16 μl of H_2_O, 0.5 μl of a reverse primer homologous to the common adaptor (stock: 10 μM), and 0.5 μl of a forward primer specific to the barcoded adaptor (stock: 10 μM). PCR amplification were as follows: initial denaturation at 95 °C for 30 s, 16 cycles of denaturation at 95 °C for 30 s, primer annealing at 62 °C for 20 s, and fragment elongation at 68 °C for 15 s, and then a final fragment elongation step at 68 °C for 5 min. A total of 8 μl of PCR product was run on a 1.5% agarose gel. Qubit™ dsDNA HS assay kit was used to measure the concentration of each DNA in GBS library. Only GBS libraries with concentrations more than 5.0 ng/μl were sequenced. A total of 30 ng of each GBS library were selected and pooled, and Sera-Mag Speed Beads were used to remove small DNA fragments, dNTP, and primers. The pooled GBS libraries (100 ng) were sequenced on an Illumina Nova platform with paired-end 150bp reads. GBS reads have been submitted to NCBI-SRA (PRJNA967088).

#### Genetic linkage map construction for *C. hongkongensis* and *C. sikamea* and QTL mapping

High-quality GBS reads of all samples were mapped to the reference genome of *C. hongkongenis* using bowtie 2 (version 2.2.3.4.3) with the following parameters: --maxins 1000 --no-discordant --no-mixed --no-unal.^70^ GATK (version 3.8) was used to do SNP calling using Haplotype Caller mode with parameters: -stand_call_conf 30 –minPruning 1.^71^^,^^72^ SNPs were filtered by GATK with parameters: -restrict Alleles To BIALLELIC -select "QD > 2.0" -select "MQ > 40.0" -select "MQRankSum > -12.5" -select "ReadPosRankSum > -8.0". After that, SNPs were filtered by vcftools (version 0.1.16) with parameters: --maf 0.01 --minDP 4 --max-missing 0.8 --min-alleles 2 --max-alleles 2.^73^

The genotype of siblings in the vcf files were transformed into JoinMap format. Parental genotypes were compared to transform their genotypes to aaxbb, hkxhk, lmxll, nnxnp etc.

Markers were removed from linkage analysis if they were not polymorphic between parents or if the missing rate exceeded more than 20% in the progeny. A chi-square test was used to test the goodness-of-fit of the observed versus expected Mendelian ratios of segregating loci. Filtered markers were employed to construct the linkage map using Joinmap 4.0.^74^ To improve efficiency and accuracy, SNPs with the same parental genotype were used. An in-house python script was written to allow for missing genotypes in some progeny.

By using Joinmap 5.0 with default parameters, markers were grouped into 10 LGs with a LOD threshold of 2-15. Both male and female maps were calculated using the Monte Carlo ML algorithm, and the consensus map was merged using MergeMap.^75^

QTL mapping for shell height and shell length was conducted by interval mapping (IM) in MapQTL 6.0.^76^ LOD scores were calculated using the interval mapping method (1 cM). By using the software MapQTL 6.0, the linkage group-wide LOD threshold and genome-wide were calculated using permutation test of 1,000 replicates.^82^^,^^83^ QTLs with LOD scores larger than LOD threshold at 95% permutation test at the linkage group-wide level were considered significant.

Based on the locations of markers, the sequences of targeted loci regions between two adjacent markers were extracted from the physical position of the *C. hongkongensis* reference genome by referring to Zhang et al.^17^

Pearson’s correlation between shell height and shell length was calculated for all progeny.

#### Identification of potential candidate genes

The positions (in base-pair) of QTL intervals were compared to the genome annotation file of *C. hongkongensis*.^17^ Gene located within these intervals were extracted and annotated using the Kyoto Encyclopedia of Genes and Genomes (KEGG) and Clusters of Gene Ontology (GO) databases.

#### Characterization and functional analysis of the *Polycystin* gene

Adult *C. hongkongensis* (approximately two-year-age; shell height 12.00 ± 1.50 cm) and adult *C. sikamea* (approximately two-year-age; shell hight 8.63 ± 1.07 cm) were purchased from Zhanjiang of Guangdong province, China. Before experiments, adult oysters were acclimated in filtered seawater with aeration (400 L tank) for at least one week under controlled temperature (25-28 °C) and salinity (15-20 ppt). Oysters were fed twice daily with *Isochrysis galbana* and *Tetraselmis suecica*.

Larvae at different developmental stages and tissues (labial palps, upper mantle, lower mantle, digestive gland, hemocytes, gill, heart, adductor muscle, and gonad) were extracted from nine adult oysters. TRIzol RNA isolation kit (Invitrogen, USA) was used to preserve larvae and tissue samples individually and stored at −80 °C for subsequent RNA isolation.

To study the expression pattern of *Polycystin* mRNA during shell repair, a shell notching experiment was conducted according to the method described by Mount et al.^49^ with some modifications. Sixty adult oysters of similar size, weight, and age were selected, and V-shaped notch was made in the shell margin near the adductor muscle in thirty individual oysters, while the remaining thirty oysters served as controls. Oysters were maintained in filtered seawater under hatchery conditions. A total of three oysters were sampled at random from each group, and mantle tissues at the notching area were collected at 0, 6, 12, 24, 36, 48, 72, 96, 120 and 144 h after shell notching. The experiment was performed in triplicate.

Based on Reduced-Representation Sequencing data of *C. gigas* and *C. hongkongensis*, the full-length cDNA of *polycystin* gene was cloned using the rapid amplification of cDNA ends (RACE) method from mantle RNA (1 μg) (Table S1).

The generated *polycystin* sequences were blasted on the National Center for Biotechnology Information (NCBI) database. The open reading frame (ORF) of *Polycystin* was identified using ORF Finder. Protein and nucleotide sequences were analyzed using BLASTX and BLASTN, respectively. The Simple Modular Architecture Research Tool (SMART) program was used to analyse the structure of proteins. Clustal W method in Bio-edit software was used to perform multiple protein sequence alignments.^77^ MEGA6.0 software was used to construct a phylogenetic tree based on the complete sequence of amino acids.^78^

Total RNA was extracted from larvae at different developmental stages and from nine tissues of three healthy adults. PCR was performed on the first-strand cDNA template. The 2^−ΔΔCT^ method was used to calculate the relative expression of *Polycystin* gene.^84^ All experiments were performed in triplicate, with *GAPDH* as an internal control.

*In-situ* hybridization (ISH) was performed to examine the spatial distribution of *Polycystin* mRNA in the mantle using a digoxigenin (DIG)-labeled antisense RNA probe synthesized using a Digoxigenin (DIG; Roche Applied Science, Penzberg, Germany) (Table S1). Adiethyl phosphocyanidate (DEPC)-treated scalpel was used to extract a small piece(0.8 × 0.5 cm) of mantle tissue from adult *C. hongkongensis*, and then fixed in 4% (w/v) paraformaldehyde/0.1 M phosphate-buffered saline (pH7.2–7.6) containing 0.1% DEPC for overnight, rinsed with 10% PBS solution for 30 min, and dehydrated in 30% sucrose solution overnight at 4 °C. The dehydrated mantle sample was then embedded in liquid nitrogen and OCT embedding agent. Tissue blocks were sectioned to 10 μm thickness and mounted on glass slides for *in situ* hybridization according to the method described by Yamaura et al.^85^

Double-stranded RNA (dsRNA)-mediated RNA interference technology (RNAi) was applied to functionally characterize the *Polycystin* gene in the shell formation process of oyster. Promega RiboMAX Express RNAi System was used to synthesis green fluorescent protein (GFP) dsRNA and *Polycystin* double-strand RNA (dsRNA) according to the manufacture instructions. The vector pEGFP-N1 (NEB, Massachusetts, USA) was used as a template to amplify EGFP using specific primers (Table S1). DNase/RNase-free water (Tian Gen) was used to dilute purified ds*Polycystin* into 100 μg/100 μL, and was then injected to the adductor muscle of oysters. Each group consisted of ten individuals of similar weight, age, and size, with a V-shaped notch cut into the shell margin of each oyster. A supplementary injection was administered three days after the first injection to enhance RNA interference efficiency.

All oysters should be sampled for mantle tissue extraction after six days of injection, and the extracted mantle tissues were preserved with TRIzol RNA isolation kit (Invitrogen, Carlsbad, USA) at -80 °C. The relative expression level of *Polycystin* in each group was determined as previously described. Meanwhile, the regenerated prismatic shells from the V-shaped notch were extracted using tweezers, marked (ds*Polycystin* group and dsEGFP group) and examined under scanning electron microscope. The relative expression level of *Polycystin* was determined as previously described, with GAPDH mRNA as internal control (Table S1). The condition of regenerated prismatic shells was examined under electron microscope (FEI Quanta 200, Holland).

### Quantification and statistical analysis

SPSS 19.0 software (SPSS Inc., USA) was used for data processing and statistical analyses. Data are expressed as means ± SD. Student’s t-tests and one-way ANOVA (followed by Turkey’s post hoc test) were used for comparisons between two groups and more than two groups, respectively. Significant differences were indicated by (*P < 0.05*) or with^∗^ (*P < 0.05*), ^∗∗^(*P < 0.01*).

## Supplemental information

Document S1. Figures S1–S3

Table S1. Oligonucleotide primers used for this study, related to STAR methods

Table S2. Values of shell height and shell length for every progeny of two hybridized familis of *Crassostrea hongkongensis* and *C. sikamea*, related to Figure 1

Table S3. Genotype of HS family, related to Figure 3

Table S4. Genotype of SH family, related to Figure 3
