# Supplementary material for: High-resolution genetic maps and QTL mapping applications reveal polycystin gene involvement in oyster shell formation
Source: iScience. 2025 Nov 10;28(12):113986. doi: 10.1016/j.isci.2025.113986 (PMC12677090; doi:10.1016/j.isci.2025.113986)
Supplement: Document S1. Figures S1–S3 [file mmc1.pdf]

iScience, Volume *28*

Supplemental information

High-resolution genetic maps and QTL mapping applications reveal *polycystin* gene involvement in oyster shell formation

Haitao Ma, Yanping Qin, Dongmei Yu, Bingke Jiao, Qingliang Liao, Yang Zhang, Yinjie Zhang, Jingyue Huang, Jun Li, Ziniu Yu, and Yuehuan Zhang

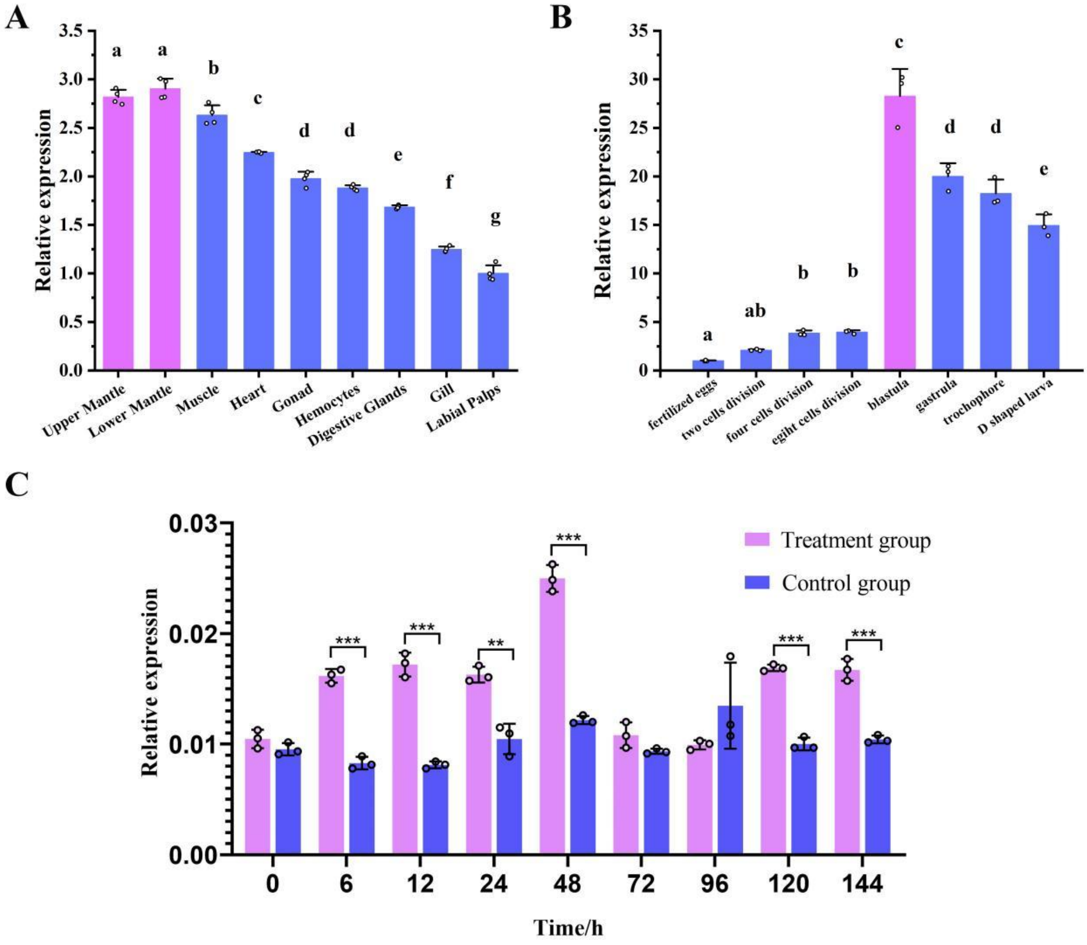

Figure S1 Expression analysis of *polycystin* by qRT-PCR in *C. sikamea*, related to Figure 7

(A) the tissue distribution of *polycystin* in different tissues, where GAPDH gene was used as a reference gene.

(B) the expression profile of *polycystin* in early developmental stages, where GAPDH gene was used as a reference gene.

(C) the temporal expression of *polycystin* after shell notching in mantle, where GAPDH gene was used as a reference gene.

Note: The significance of data was analyzed using one-way ANOVA (for **A** and **B**) and *t*-test (for **C**) by the SPSS software. The dots represent biological replicates and the vertical bars represent the mean ± SD (N = 3). In the same graph, different letters indicate significant differences ($P < 0.05$). ** and *** indicate a significant difference at $P < 0.01$ and $P < 0.001$, respectively, at the same point in time.

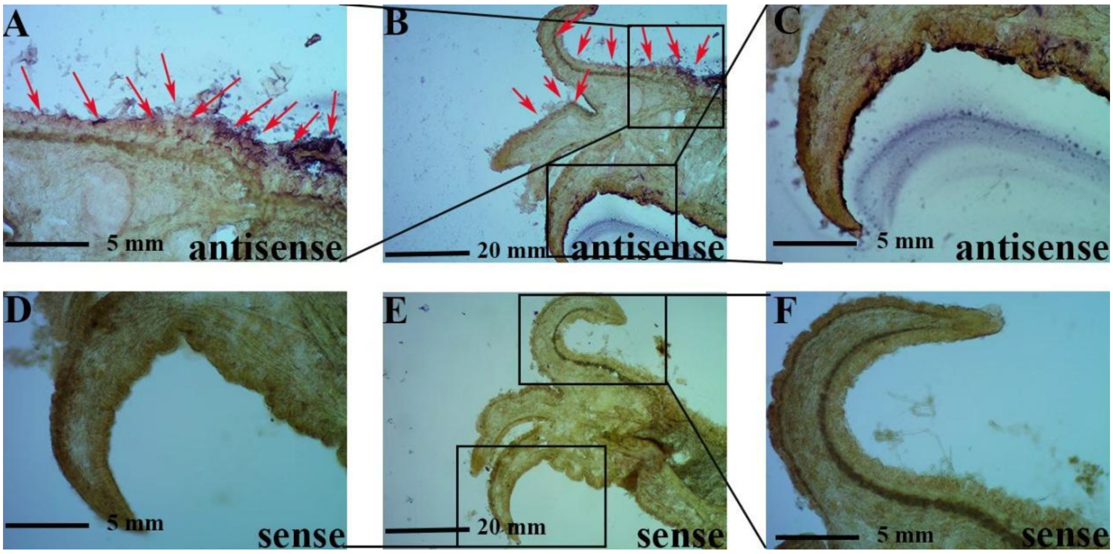

Figure S2 *In-situ* hybridization of *polycystin* in mantle tissue of *C. sikamea*, related to Figure 8

The red arrows indicate the positive signal. A positive signal is indicated by the blue color.

(A, **B** and **C)** the picture of experimental group. The red arrows are indicated the positive signal.

(D, **E** and **F)** the picture of control group.

In **A**, **C**, **D** and **F**, bars = 5 mm. In **B** and **E**, bars = 20 mm.

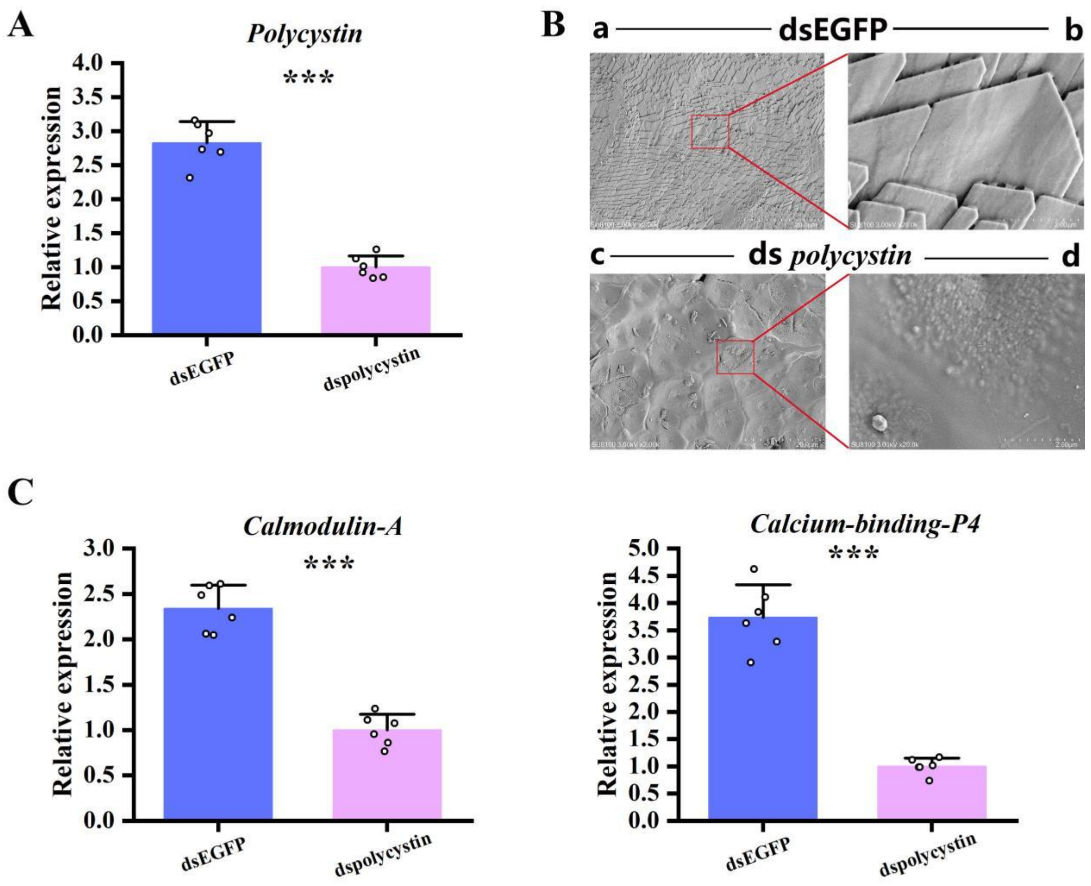

Figure S3 RNA interference of *polycystin* in *C. sikamea*, related to Figure 9

(A) The expression of *polycystin* was determined 6 days after 100μg dsRNA injection by q-PCR, where GAPDH gene was used as a reference gene.

(B) SEM images of prismatic layer after injecting dsRNA. In **a** and **c**, bars = 20.0 μm. In **b** and **d**, bars = 2.00 μm.

(C) Relative expression levels of Calmodulin-A, Calcium-binding-P4 after RNAi, where GAPDH gene was used as a reference gene.

Note: The dots represent biological replicates and the vertical bars represent the mean ± SD (N = 6, biological replicates). The significance of data was analyzed using *t*-test by the SPSS software. *** indicates a significant difference at *P* < 0.001 between the ds EGFP groups and ds *polycystin* groups.
